# Supplementary material for: A Small-Particle Aerosol Model of Ebolavirus Zaire Infection in Ferrets
Source: Viruses. 2024 Nov 21;16(12):1806. doi: 10.3390/v16121806 (PMC11680438; doi:10.3390/v16121806)
Supplement: Supplementary file 1 [file viruses-16-01806-s001.zip › viruses-3198039-supplementary.pdf]

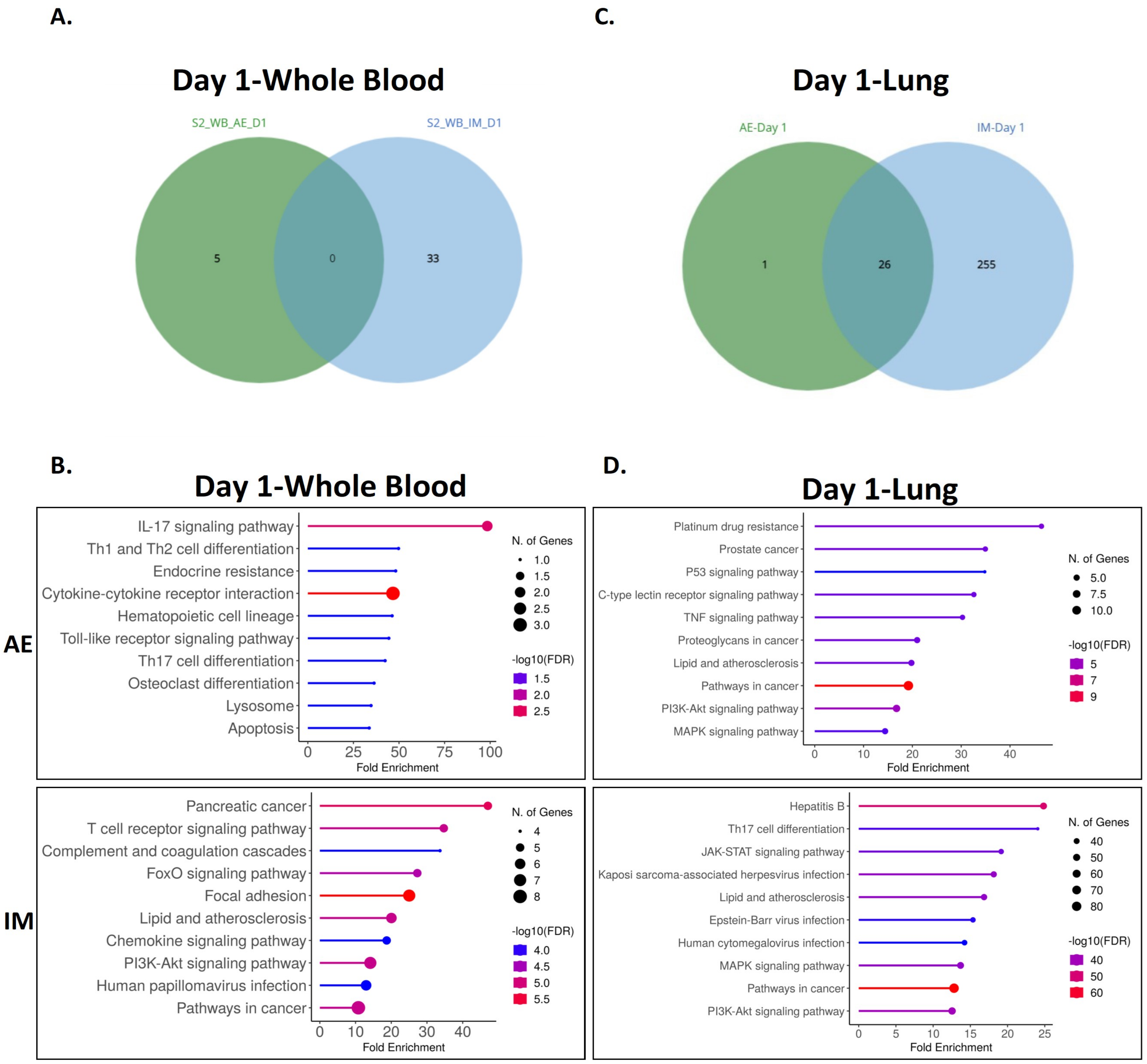


**Supplementary Figure 1. Transcriptomic analysis of whole blood and lung 1 day post-EBOV exposure.** Whole blood analysis at 1 dpi of (A) Challenge route-associated overlap of DEGs at 1 dpi, and (B) KEGG pathway analysis of significantly enriched pathways. Lung homogenate analysis at 1 dpi of (C) Challenge-route associated overlap of DEGs, and D) KEGG pathway analysis of significantly enriched pathways. All of the above differential analysis is based on aggregate analysis of 4 ferrets per group time point.

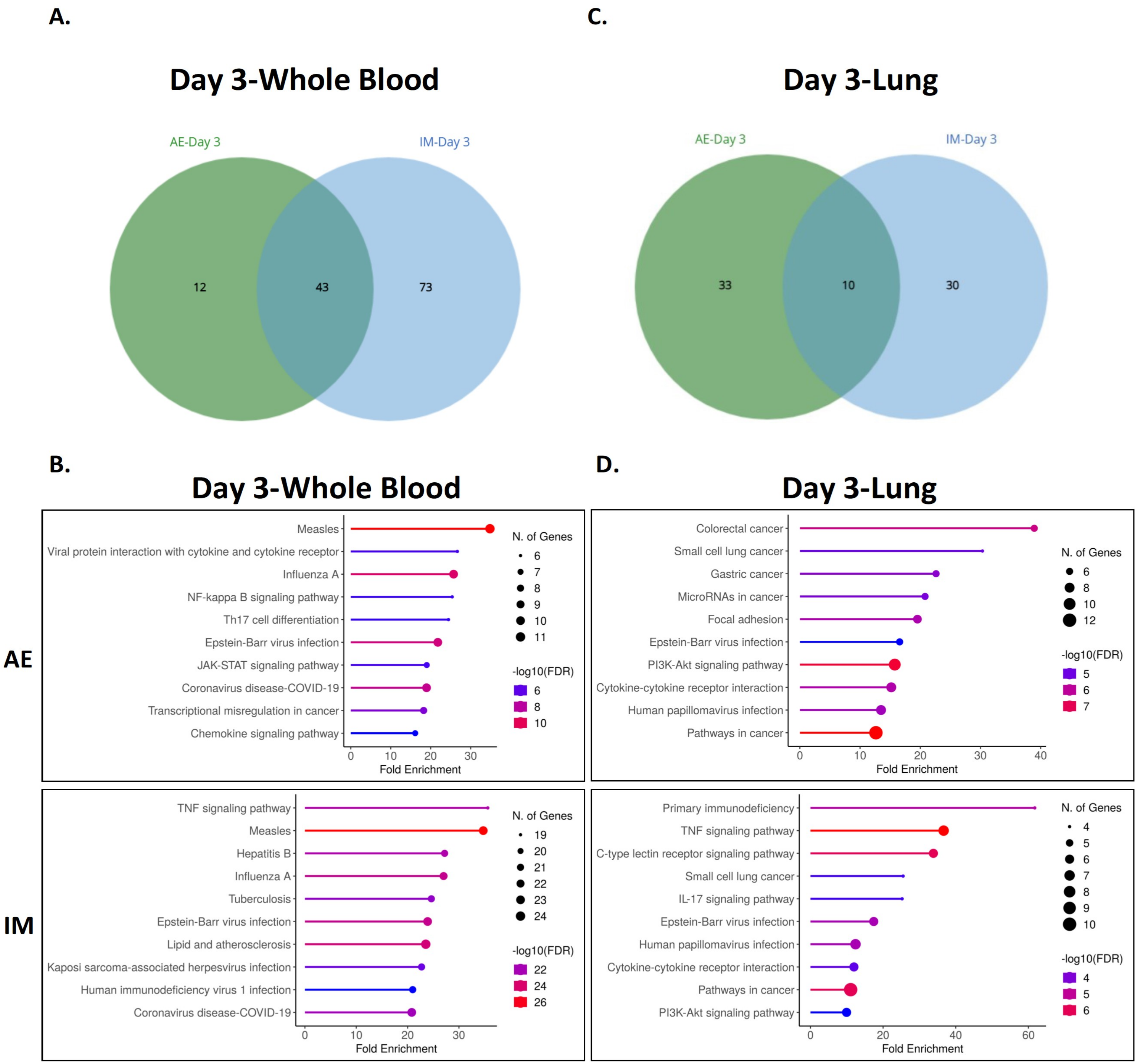


**Supplementary Figure 2. Transcriptomic analysis of whole blood and lung 3 day post-EBOV exposure.** Whole blood analysis at 3 dpi of (A) Challenge route-associated overlap of DEGs, and (B) KEGG pathway analysis of significantly enriched pathways. Lung homogenate analysis at 3 dpi of (C) Challenge-route associated overlap of DEGs, and D) KEGG pathway analysis of significantly enriched pathways. All of the above differential analysis is based on aggregate analysis of 4 ferrets per group time point.

**Supplementary Table 1. Significant differentially expressed mRNAs in ferret whole blood, 1 dpi.**

| | IM | | AE | |
|---|---|---|---|---|
| | **Gene** | **$Log_2$ Diff** (*p value*) | **Gene** | **$Log_2$ Diff** (*p value*) |
| **Upregulated** | SMAD3 | 5.13 *(0.0065)* | CD68 | 4.46 *(0.0024)* |
| | C1QBP | 4.70 *(0.0018)* | IL17RB | 4.46 *(0.038)* |
| | CCR3 | 4.13 *(0.024)* | IL1RL1 | 3.46 *(0.026)* |
| | NLRP3 | 4.05 *(0.0022)* | _ | _ |
| | NOX1 | 3.64 *(0.030)* | _ | _ |
| | ATG10 | 3.46 *(0.0055)* | _ | _ |
| | RFXAP | 3.17 *(0.047)* | _ | _ |
| | PTPN22 | 2.36 *(0.0011)* | _ | _ |
| | CASP7 | 2.04 *(0.0074)* | _ | _ |
| | CDC42 | 1.49 *(0.0072)* | _ | _ |
| **Downregulated** | CD55 | -1.94 *(0.0039)* | CSF3R | -2.35 (*0.0072*) |
| | EGR1 | -2.04 *(0.045)* | FOS | -5.34 (*0.011*) |
| | FANCD2 | -2.29 *(0.0038)* | _ | _ |
| | VWF | -2.37 *(0.0057)* | _ | _ |
| | GP6 | -2.48 *(0.00052)* | _ | _ |
| | THBS1 | -4.22 *(0.00081)* | _ | _ |
| | IGF1 | -5.36 *(0.0014)* | _ | _ |
| | CXCL6 | -6.15 *(0.00031)* | _ | _ |
| | EGF | -7.69 *(0.00077)* | _ | _ |
| | CLU | -7.93 *(0.020)* | _ | _ |

Top 10 of most upregulated and downregulated genes included. Inclusion criteria were a linear 2-fold difference and p-value $\leq$ 0.05.

**Supplementary Table 2. Significant differentially expressed mRNAs in ferret whole blood, 3 dpi.**

| | IM | | AE | |
|---|---|---|---|---|
| | **Gene** | **$Log_2$ Diff** (*p-value*) | **Gene** | **$Log_2$ Diff** (*p-value*) |
| **Upregulated** | IDO1 | 8.42 *(0.018)* | IDO1 | 7.92 *(0.030)* |
| | DHX58 | 8.11 *(0.012)* | IL27 | 6.90 *(0.041)* |
| | IL27 | 7.97 *(0.014)* | CCL8 | 6.08 *(0.046)* |
| | OASL | 7.34 *(0.036)* | IFIT1 | 4.89 *(0.011)* |
| | ISG15 | 5.56 *(0.0034)* | ISG15 | 4.68 *(0.018)* |
| | IFIT1 | 5.22 *(0.0047)* | IFIH1 | 4.56 *(0.012)* |
| | MX1 | 5.17 *(0.0057)* | MX1 | 4.42 *(0.020)* |
| | CLEC7A | 5.10 *(0.0107)* | IFIT3 | 4.27 *(0.011)* |
| | SOCS1 | 4.93 *(0.0017)* | DDX58 | 3.90 *(0.014)* |
| | IFIH1 | 4.70 *(0.0050)* | SOCS1 | 3.76 *(0.030)* |
| **Downregulated** | CD4 | -1.58 *(0.00052)* | FYN | -1.095 *(0.023)* |
| | EPOR | -1.74 *(0.0046)* | IL6ST | -1.15 *(0.013)* |
| | LCK | -1.75 *(0.0014)* | CD3E | -1.23 *(0.022)* |
| | NPC1 | -1.85 *(0.030)* | IL2RB | -1.30 *(0.0038)* |
| | PRF1 | -2.05 *(0.046)* | CCND2 | -1.33 *(0.022)* |
| | VTN | -2.078 *(0.021)* | TRIM33 | -1.34 *(0.0011)* |
| | PLCG1 | -2.30 *(0.013)* | CD5 | -1.56 *(0.0029)* |
| | LTA | -3.21 *(0.039)* | KLRD1 | -1.60 *(0.0041)* |
| | C8A | -4.09 *(0.011)* | CD84 | -1.61 *(0.0051)* |
| | FOS | -6.60 *(0.0026)* | CCR7 | -1.69 *(0.0075)* |

Top 10 of most upregulated and downregulated genes included. Inclusion criteria were a linear 2-fold difference and p-value $\leq$ 0.05.

**Supplementary Table 3. Significant differentially expressed mRNAs in ferret lung homogenate, 1 dpi.**

| | IM | | AE | |
|---|---|---|---|---|
| | **Gene** | **$Log_2$ Diff (*p-value*)** | **Gene** | **$Log_2$ Diff (*p-value*)** |
| **Upregulated** | IL17B | 4.48 (*0.0067)* | COL1A1 | 3.71 *(0.00071)* |
| | IFNL1 | 4.22 *(0.0082)* | IFNL1 | 3.20 *(0.044)* |
| | GAST | 3.64 *(0.040)* | IGF2 | 2.64 *(0.044)* |
| | POMC | 3.47 *(0.015)* | RPLP0 | 1.41 *(0.0091)* |
| | PTX3 | 3.26 *(0.0052)* | _ | _ |
| | TCF3 | 3.25 *(0.0078)* | _ | _ |
| | CD4 | 3.038 *(0.018)* | _ | _ |
| | IGF2 | 2.91 *(0.027)* | _ | _ |
| | C8A | 2.80 *(0.032)* | _ | _ |
| | C1QB | 2.74 *(0.038)* | _ | _ |
| **Downregulated** | PIK3C3 | -6.98 *(0.00013)* | FANCD2 | -3.91 *(0.029)* |
| | NFATC3 | -7.25 *(0.0029)* | IFNLR1 | -3.95 *(0.013)* |
| | MFGE8 | -7.57 *(0.0013)* | MMP9 | -4.13 *(0.035)* |
| | PTPN2 | -7.59 *(6.68E-05)* | IL17RD | -4.49 *(0.020)* |
| | ABL1 | -7.65 *(0.018)* | ATG12 | -4.52 *(0.029)* |
| | P2RX7 | -7.68 *(0.00077)* | IKBKG | -4.73 *(0.019)* |
| | PRKDC | -7.69 *(0.018)* | IGF1R | -4.82 *(0.018)* |
| | PLD1 | -8.047 *(0.00069)* | PMAIP1 | -5.11 *(0.018)* |
| | IL6ST | -8.43 *(0.00010)* | MDM2 | -5.70 *(0.015)* |
| | PDGFRA | -8.51 *(0.0072)* | CFH | -6.24 *(0.0073)* |

Top 10 of most upregulated and downregulated genes included. Inclusion criteria were a linear 2-fold difference and p-value $\leq$ 0.05.

**Supplementary Table 4. Significant differentially expressed mRNAs in ferret lung homogenate, 3 dpi.**

| | IM | | AE | |
|---|---|---|---|---|
| | **Gene** | **$Log_2$ Diff** *(p-value)* | **Gene** | **$Log_2$ Diff** *(p-value)* |
| **Upregulated** | ISG15 | 5.14 *(0.0052)* | CCL4 | 6.62 *(0.016)* |
| | IL17B | 4.27 *(0.0097)* | CCL5 | 5.73 *(0.049)* |
| | GAST | 3.91 *(0.028)* | MX1 | 4.79 *(0.019)* |
| | IGF2 | 3.75 *(0.0043)* | CCL22 | 4.28 *(0.025)* |
| | IFNL1 | 3.72 *(0.020)* | CXCL10 | 4.12 *(0.038)* |
| | POMC | 3.32 *(0.020)* | CCL8 | 3.77 *(0.042)* |
| | COL1A1 | 3.13 *(0.0039)* | CDKN1A | 3.53 *(0.041)* |
| | TCF3 | 2.96 *(0.015)* | PTX3 | 2.90 *(0.012)* |
| | CD19 | 2.59 *(0.033)* | IL1RN | 2.80 *(0.030)* |
| | PTX3 | 2.59 *(0.025)* | COL1A1 | 2.47 *(0.020)* |
| **Downregulated** | IL23A | -3.392 *(0.047)* | FLT1 | -1.88 *(0.026)* |
| | PTPN22 | -3.555 *(0.036)* | HGF | -2.04 *(0.033)* |
| | FCER1A | -3.807 *(0.044)* | GHR | -2.06 *(0.050)* |
| | ATG10 | -3.845 *(0.038)* | ENTPD1 | -2.20 *(0.013)* |
| | FANCD2 | -3.907 *(0.029)* | PDGFRB | -2.30 *(0.037)* |
| | MMP9 | -4.129 *(0.035)* | VEGFC | -2.54 *(0.039)* |
| | IL17RD | -4.492 *(0.020)* | PTGS2 | -2.87 *(0.029)* |
| | ATG12 | -4.524 *(0.029)* | ATG10 | -3.68 *(0.046)* |
| | IKBKG | -4.728 *(0.019)* | CCL24 | -4.17 *(0.016)* |
| | HMGB1 | -5.285 *(0.0063)* | IL17RD | -4.49 *(0.020)* |

Top 10 of most upregulated and downregulated genes included. Inclusion criteria were a linear 2-fold difference and p-value $\leq$ 0.05.
